# Training Pediatric Residents to Provide Smoking Cessation Counseling to Parents

**DOI:** 10.1100/tsw.2005.54

**Published:** 2005-05-13

**Authors:** Rebecca L. Collins, Sandy D'Angelo, Sarah D. Stearns, Lynn R. Campbell

**Affiliations:** ^1^ Department of Pediatrics, University of Kentucky Chandler Medical Center, Lexington, KY, USA; ^2^ Behavioral Sciences, University of Kentucky Chandler Medical Center, Lexington, KY, USA; ^3^ Behavioral Sciences Department, La Rabida Children's Hospital, Chicago, IL, USA

**Keywords:** human development, public health, smoking, prevention, United States

## Abstract

The objective was to assess the effectiveness of a smoking cessation educational program on pediatric residents' counseling. Residents were randomly selected to receive the intervention. Residents who were trained were compared to untrained residents. Self-reported surveys and patient chart reviews were used. Measures included changes in self-reported knowledge, attitudes and behaviors of residents, and differences in chart documentation and caretaker-reported physician counseling behaviors. The intervention was multidimensional including a didactic presentation, a problem-solving session, clinic reminders, and provision of patient education materials. Results showed that residents who were trained were more likely to ask about tobacco use in their patients' households. They were also more likely to advise caretakers to cut down on or to quit smoking, to help set a quit date, and to follow up on the advice given at a subsequent visit. Trained residents were more likely to record a history of passive tobacco exposure in the medical record. These residents also reported improved confidence in their counseling skills and documented that they had done such counseling more often than did untrained residents. Caretakers of pediatric patients who smoke seen by intervention residents were more likely to report that they had received tobacco counseling. Following this intervention, pediatric residents significantly improved their behaviors, attitudes, and confidence in providing smoking cessation counseling to parents of their pediatric patients.

### INTRODUCTION

Smoking is the leading preventable cause of death in our country[1]. Since pediatricians do not deal directly with the long-term effects of tobacco use, they may feel less responsible for counseling adults who smoke. However, pediatricians are frequently challenged with the ill effects of passive tobacco exposure on children. Recent statistics show that 38% of U.S. children younger than 6 years of age are exposed to environmental tobacco smoke (ETS) in their homes[2]. The detrimental effects of ETS have been well documented and include an increased risk of wheezing, asthma, chronic bronchitis, chronic otitis media requiring surgical tympanostomy placement, and sudden infant death syndrome[2,3].

As a result of the increasing body of evidence regarding the negative effects of ETS on young children, pediatricians are being urged to take a more active role in counseling caretakers who smoke. The American Academy of Pediatrics policy statement regarding ETS recommends that pediatricians take a smoking history from parents, inform parents about the health hazards of passive smoking, and provide guidance on smoking cessation[3]. Despite these recommendations, a recent study documented that pediatricians report that they are less likely to discuss smoking cessation with parents who smoke than with their adolescent patients[4]. Furthermore, pediatricians obtain a caretaker smoking history only 40% of the time and record it in the medical chart only 11% of the time[5]. When compared to other physicians, pediatricians were significantly less likely than family practice physicians to counsel and intervene with parents who smoke[6]. One explanation for this may be that pediatricians lack sufficient training in smoking cessation counseling (SCC)[6,7].

Practicing pediatricians report that they are more likely to do SCC if they have had formal training[4]. Residency is the ideal place for this training to occur. Several interventional studies have been conducted in attempts to improve resident knowledge, skills, and confidence in SCC[7,8,9,10,11,12,13]. The majority of residents involved in these studies were Internal Medicine and Family Practice residents who were encouraged not only to counsel, but also to offer treatment to their patients who smoke.

Three previous studies examined the effect of SCC training on resident self-reports of SCC behavior in pediatric residency settings[8,12,13]. While two of these studies used caretaker interviews to validate the self-reports[8,12], none compared resident behaviors and attitudes to a control group of residents working in the same clinic who did not receive the SCC training. Therefore, the purpose of this study was to assess the effectiveness of a brief educational program on SCC for pediatric residents in a university-based continuity clinic in comparison to a group of residents who did not receive the intervention and to validate any self-reported changes in SCC behaviors by independent, objective measures.

### METHODS

Residents participating in pediatric continuity clinics were chosen to receive either SCC training or an educational program unrelated to SCC. The two groups were compared for SCC behaviors and attitudes pre- and postintervention. In addition, parents who smoke were surveyed to validate SCC efforts of the two resident groups.

#### Setting and Study Subjects

Resident subjects were recruited from existing continuity clinic teams at the University of Kentucky Medical Center. Continuity clinic teams include residents in Categorical Pediatrics as well as residents in two combined programs: Internal Medicine and Pediatrics (Med-Peds) and Child and Adult Psychiatry and Pediatrics (Peds-Psych). Residents attending clinic on Monday and Wednesday were designated as control residents. The residents attending clinic on the three other clinic half-days were given the educational intervention at preclinic conference and, thus, became the intervention residents. All 35 residents assigned to pediatric continuity clinic were asked to participate in the study. Two declined; both attended clinic on control days. Thus 33 residents (21 in the intervention group and 12 in the control group) participated in the study. A total of 656 caretakers of pediatric patients who were seen by these residents for scheduled continuity clinic visits during a 6-month period also participated in this study. The study protocol (#95-00253) was approved by the Institutional Review Board and the Committee on the Protection of Human Subjects of the University of Kentucky.

### Materials, Interventions, and Measures

A preintervention, self-report survey was obtained from all participating residents. This survey assessed baseline demographic information and included nonvalidated questions measuring the residents' knowledge, attitudes, and behaviors with regard to SCC, as well as perceived barriers to such counseling. The intervention residents then received a multidimensional, educational intervention including a didactic presentation based on the National Cancer Institute's Smoking Cessation Training Program[14,15] with an accompanying written manual, a small group problem-solving session, written reminders about counseling that were placed in the patient rooms and general work areas on clinic days, and provision of written educational and self-help materials regarding smoking cessation for distribution to caretakers.

The didactic lecture and small group session, conducted by the principle investigator (RLC) who is a general pediatrician, occurred at two required preclinic conferences over a 1.5-h period. All of the 21 intervention residents attended the didactic lecture on smoking cessation, 19 participated in the small group teaching session, and 20 reported that they received the NCI Training Manual. The faculty preceptors for the continuity clinics designated as intervention days also attended the didactic conference. Residents and faculty attending continuity clinic on control days had alternative didactic instruction unrelated to SCC. Clinic reminders were not displayed on control days, and, though patient education materials were always available if requested by the resident, these materials were placed with other educational materials and were not conspicuously available. After the 6-month data-collection period, all of the participating residents completed a second self-report survey that was identical to the one given prior to the educational intervention

The records of all scheduled patients were stamped with an obvious "Is there a smoker in the home?" reminder at the time of registration. Caretakers were asked to complete a questionnaire regarding their smoking behaviors and knowledge, as well as their attitudes toward SCC. Nurses recorded information regarding environmental tobacco exposure and collected the caretaker questionnaires prior to the physician encounter with the parent. Chart reviews were completed on all patients whose caretakers identified themselves as current smokers to monitor documentation of an elicited smoking history, SCC, and distribution of smoking cessation literature.

To validate the self-reported physician-counseling behavior, a telephone survey was performed 1–2 weeks following the pediatric visit. This standardized telephone interview was conducted with consenting caretakers who were present at the visit to establish whether the physician had discussed smoking during the visit and to determine what advice was offered. Individuals who were blind to resident group assignment conducted the telephone interviews.

### Analysis

Resident and caretaker groups were compared using unpaired t-test and contingency table analyses. Variable means were calculated for the intervention and control groups. Pre- and postintervention resident surveys were compared using paired statistical testing. Statistical comparisons were made using t-tests for continuous variables and $\chi^2$ tests for nominal variables, with a two-tailed significance level of $p = 0.05$. The results that follow report on the data collected from 21 intervention residents, 12 control residents, and the 656 caretakers seen by these residents over the 6-month study period.

## RESULTS

### Preintervention Demographics and SCC Variables

All but 1 of the 33 participating residents completed the baseline survey. Residents provided the educational intervention were similar to the control residents with regard to gender and level of training, although they were somewhat younger (mean age 28.8 vs. 30.5 years, $p < 0.01$); 62% of the participants were male, 35% were in their first year of training, and 21% had smoked tobacco in the past, although none of them smoked or lived with a smoker at the time of the study. The majority of residents reported they had received prior training in smoking cessation in medical school and approximately one-quarter acknowledged a previous lecture during their residency. More than half of the residents had done self-reading in an attempt to learn more about smoking cessation. Nearly all (97%) stated they would be willing to learn brief smoking cessation techniques to be used in the care of their pediatric patients' caretakers. Experimental and control residents did not differ in any baseline demographic attribute except age.

All of the residents completed a 17-item true/false test to assess their baseline knowledge regarding the adverse health effects of tobacco use and exposure. The residents demonstrated good baseline knowledge with an average score of 13.3 of 17 correct (78%). There was no difference in baseline knowledge between the residents who were to receive the intervention and those who were not. They were also asked to report how often they practiced 15 different smoking cessation behaviors in counseling using a 5-point scale ranging from "hardly ever" to "almost all of the time". At baseline, there were no differences between the intervention and the control residents with regard to their self-reported practices of SCC behaviors as shown in Table 1. The vast majority of the residents (91%) reported that they spent between 1 and 5 min counseling on smoking issues.

Residents were also asked to rate (on a 5-point scale) the attitudes they had regarding tobacco counseling by pediatricians and the barriers they perceived in providing such counseling. As shown in Table 1, both intervention and control residents thought it was more important for pediatricians to counsel about the adverse consequences of passive tobacco exposure than the dangers of smoking to the smoker. Resident groups reported equal confidence in their counseling skills and willingness to distribute educational materials to smoking caretakers. The groups were also similar in their perceptions of barriers to providing such counseling, though the control residents were somewhat more likely to think that the caretakers have too many other (more urgent) problems that make tobacco smoking less of a priority. Both groups thought the greatest barriers of SCC were "limitations of time to provide such counseling" and "caretaker resistance in discussing the topic".

Demographic information regarding the 656 caretaker participants was obtained through a baseline survey. The caretakers were primarily Caucasian mothers who averaged 28.9 years and had at least a high school education. Almost half of these caretakers worked outside of the home and 47% reported a spouse as their source of primary support. Nearly half reported that there was a smoker in the home and 40% reported that they personally had smoked regularly at some time in the past. At the time of the study, 29% (188) reported that they smoked at least five cigarettes a day. Among the caretakers who smoked, roughly one-third had smoked for more than 10 years, 40% had started smoking in early adolescence, and the majority currently smoked at least half a pack per day. Half of them planned not to be smoking in 5 years and 74% had tried to quit smoking at some time in the past. Of note, more than one-third (39%) reported that they would like to quit sometime in the next 6 months and 62% reported that they felt that their children would be healthier if they quit smoking. There were no differences in demographic caretaker attributes between those who were seen by intervention residents and those seen by control residents.

Of the caretakers who smoked, 40% thought it was appropriate for their pediatricians to discuss their smoking behaviors with them and more than a third (35%) thought that they would like their children's doctors to help them quit smoking. Almost half (45%) reported that they had been asked about their smoking histories by their pediatricians at some time in the past. Of the caretakers who smoked, 30% reported that they had a personal physician (other than an OB-GYN), but only 28% had seen their doctors in the last year. These findings were similar among the caretakers who smoke in the intervention and control groups. Slightly more than half (52%) of these caretakers agreed to a follow-up telephone call in 1 week to review the preventive counseling that had been done at the visit.

**TABLE 1**
**Residents' Baseline SCC Behaviors, Attitudes, and Barriers**

| | Intervention (n = 20) | Control (n = 12) | *p*-Value[a] |
|---|---|---|---|
| **Reported behaviors[b]** | | | |
| Asks about tobacco at first visit | 3.15 | 3.5 | 0.54 |
| Asks at subsequent visits | 3.1 | 2.67 | 0.28 |
| Asks about other smokers in home | 3.4 | 3.17 | 0.64 |
| Asks about smokers at daycare | 2 | 2.33 | 0.45 |
| Records tobacco exposure on problem list | 1.7 | 1.58 | 0.75 |
| Discusses smoking risks to smoker | 3.2 | 3.08 | 0.83 |
| Discusses risks of passive exposure | 4.05 | 3.75 | 0.44 |
| Advises change in location | 4.1 | 4.25 | 0.68 |
| Advises cutting down | 3.25 | 3 | 0.64 |
| Advises smoker to quit | 3.4 | 3.5 | 0.83 |
| Helps set a quit date | 1.55 | 2.17 | 0.15 |
| Provides literature to smoker | 1.1 | 1.5 | 0.15 |
| Refers smoker to programs | 1.2 | 1.5 | 0.17 |
| Prescribes nicotine replacement | 1.15 | 1.33 | 0.37 |
| Follows up at later visit | 2.2 | 2.33 | 0.78 |
| **Reported attitudes[c]** | | | |
| Should counsel smoker regarding self-risk | 4.15 | 3.75 | 0.32 |
| Should counsel about passive risks | 4.9 | 4.33 | 0.1 |
| Should assist parent to stop smoking | 3.75 | 3.58 | 0.65 |
| Confidence in counseling skills | 3 | 2.75 | 0.59 |
| Willingness to distribute materials | 4.6 | 4 | 0.16 |
| **Reported barriers[d]** | | | |
| Fears alienating parent | 2.65 | 2.42 | 0.52 |
| Has limited expertise in counseling | 2.9 | 2.67 | 0.6 |
| Language or cultural differences | 1.8 | 1.67 | 0.69 |
| Not enough time to counsel | 3.15 | 3.25 | 0.81 |
| Too many other problems | 2.25 | 2.92 | 0.07 |
| Doubt that counseling works | 2.75 | 2.67 | 0.84 |
| Parents too resistant to discuss | 3.05 | 2.75 | 0.41 |

[a] Paired t-test, two tailed, significance level of $p = 0.05$.
[b] Mean for group on 5-point scale ranging from 1 = Hardly Ever to 5 = Almost All of the Time.
[c] Mean for group measured as agreement with the statement on 5-point scale ranging from 1 = Not at All to 5 = A Great Extent.
[d] Mean for group on 5-point scale ranging from 1 = Not a Barrier to 5 = Very Much a Barrier.

### Postintervention SCC Behaviors and Attitudes

Following the 6-month period of data collection, all of the residents completed a second (identical) self-assessment of their behaviors, attitudes, and beliefs regarding SCC. Means were calculated for the intervention group and the control group for each variable. The mean for each variable was compared with those obtained prior to the intervention and data-collection period with paired statistical testing. As shown in Table 2, the intervention residents reported statistically significant increases in many SCC behaviors following the intervention. These increases included ascertaining smoking histories at both the

**TABLE 2**
**Resident Self-Reported SCC Behaviors, Attitudes, and Barriers**

| | Intervention Group (n = 20) | | | Control Group (n = 12) | | |
|---|---|---|---|---|---|---|
| | Baseline | After 6 Months | *p*-Value[a] | Baseline | After 6 Months | *p*-Value[a] |
| **Reported behaviors**[b] | | | | | | |
| Asks about tobacco use at first visit | 3.15 | 4.29 | < 0.01 | 3.5 | 3.58 | 0.88 |
| Asks at subsequent visits | 3.1 | 3.76 | 0.03 | 2.67 | 2.92 | 0.57 |
| Asks about other smokers in home | 3.4 | 4.14 | 0.02 | 3.16 | 3.33 | 0.69 |
| Records family's tobacco use on problem list | 1.7 | 3.52 | < 0.01 | 1.58 | 1.92 | 0.34 |
| Offers info about passive exposure risks | 4.05 | 4.43 | 0.11 | 3.75 | 4.17 | 0.05 |
| Advises caretaker to cut down | 3.25 | 4.14 | 0.01 | 3 | 3.17 | 0.67 |
| Advises caretaker to quit | 3.4 | 4.29 | < 0.01 | 3.5 | 3.67 | 0.5 |
| Helps caretaker set a quit date | 1.55 | 2.81 | < 0.01 | 2.17 | 2 | 0.67 |
| Provides self-help literature | 1.1 | 3.05 | < 0.01 | 1.5 | 1.58 | 0.59 |
| Follows up at later visit | 2.2 | 3.57 | < 0.01 | 2.33 | 2.25 | 0.84 |
| **Reported attitudes**[c] | | | | | | |
| Confidence in counseling skills | 3 | 3.9 | < 0.01 | 2.75 | 3.5 | 0.1 |
| **Reported barriers**[d] | | | | | | |
| Fear of alienating caretakers | 2.65 | 2.05 | 0.02 | 2.42 | 2 | 0.32 |
| Limitations in counseling expertise | 2.9 | 2 | < 0.01 | 2.67 | 2.58 | 0.79 |
| Not enough time to counsel | 3.15 | 3.43 | 0.53 | 3.25 | 3.08 | 0.5 |

[a] Paired t-test, two tailed, significance level of $p = 0.05$.
[b] Mean for group on 5-point scale ranging from 1 = Hardly Ever to 5 = Almost All of the Time.
[c] Mean for group measured as agreement with the statement on 5-point scale ranging from 1 = Not at All to 5 = A Great Extent.
[d] Mean for group on 5-point scale ranging from 1 = Not a Barrier to 5 = Very Much a Barrier.

first (Δ mean + 1.14) and subsequent visits (Δ mean + 0.66), and asking whether there were other smokers in the home (Δ mean + 0.74). Intervention residents also reported that they were more likely to record the family's smoking history on the child's problem list (Δ mean + 1.82) and to follow up on their counseling advice at a later visit (Δ mean + 1.37). Further, they reported they were now more likely to provide self-help literature to the caretaker and to advise caretakers to change where they smoke, to cut down on their smoking, to quit smoking, or to set a quit date. The intervention residents also reported significant gains in confidence in counseling skills following SCC training (Δ mean + 0.9), but no other attitudinal changes. Furthermore, they also reported significant declines in their perception of "fear of alienating the caretaker" (Δ mean – 0.6) and "limitations in counseling expertise" (Δ mean – 0.9) as barriers. Though intervention residents were now more likely to report that limitations in time available to do such counseling as a barrier (Δ mean + 0.28), this did not reach statistical significance. In contrast, control residents reported no changes in their attitudes toward SCC or their perception of the barriers in providing SCC over the 6-month study period, and the only reported SCC behavior that changed in a statistically significant way was an increased frequency of counseling about the risks of passive tobacco exposure (Δ mean + 0.42).

Chart review data confirmed the increase in SCC behaviors self-reported by the intervention residents. They were more likely to document a history of smoking on the problem list (31 vs. 15%, $p$ = 0.05) or elsewhere in the chart (40 vs. 20%, $p < 0.01$) and to document that tobacco counseling had been done at the visit (36 vs. 19%, $p$ = 0.05) as compared to the control residents. In addition, as shown in Table 3, they were also more likely to document the readiness stage of the smoker toward quitting (36 vs. 17%, $p$ = 0.02) and whether the caretaker had been given patient education self-help materials at the visit (23 vs. 3%, $p < 0.01$).

**TABLE 3**
**Chart Review of Physician SCC Behavior**

| | Caretakers Who Smoke Seen by Intervention Group (n = 126) | Caretakers Who Smoke Seen by Control Group (n = 59) | *p*-Value |
|---|---|---|---|
| Documentation of ETS on problem list | 31% | 15% | 0.05 |
| Documentation of ETS elsewhere in chart | 40% | 20% | < 0.01 |
| Documentation that counseling was done | 36% | 19% | 0.05 |
| Documentation of a quit date | 6% | 2% | 0.35 |
| Documentation of readiness stage | 36% | 17% | 0.02 |
| Documentation that caretaker given written info | 23% | 3% | < 0.01 |

ETS = Environmental tobacco smoke.

The self-reports of increased counseling behaviors among the intervention residents were also confirmed by the follow-up telephone call as shown in Table 4. Of caretakers who were current smokers, 42% were reached and surveyed by telephone 1 week following their clinic visit. Caretakers reported more frequently that they had received tobacco counseling if seen by intervention residents than if seen by control residents (77 vs. 52%, $p$ = 0.02).

**TABLE 4**
**Caretaker Report of Residents' Counseling Behavior**

| | Caretakers Who Smoke Seen by Intervention Group (n = 126) | Caretakers Who Smoke Seen by Control Group (n = 59) |
|---|---|---|
| **Completed survey 1 week post visit** | 42% | 42% |
| **Parental report of counseling** | | |
| Received counseling, safety | 60% | 64% |
| Received counseling, feeding | 66% | 84% |
| Received counseling, sleeping | 53% | 72% |
| Received counseling, tobacco | 77% | 52% |

### DISCUSSION

We have demonstrated that a brief educational intervention, coupled with the use of clinic reminders and patient educational materials delivered to pediatric continuity residents can change residents' SCC behaviors in a pediatric clinic. Three previous studies reported on the impact of a tobacco education intervention in pediatric residency programs. Our data are consistent with reports by Klein et al.[12] and Kosower et al.[13] who previously showed that an educational intervention can significantly improve residents' confidence in their counseling skills and can increase the frequency with which residents report tobacco counseling.

Our data are also consistent with Hymowitz et al.[8] and Klein et al.[12] who confirmed residents' self-reported increases in SCC by parent exit interviews following a SCC intervention study. Hymowitz demonstrated significant improvements in pediatric residents' behavior regarding SCC after a comprehensive, 12-h training program and verified the changes by parents' reports, but used residents in two other programs (Internal Medicine and Psychiatry) as controls.

Our study confirmed the benefits of resident training in SCC with an experimental design that included a control group of residents in the same pediatric clinic who did not receive the intervention. The importance of the control group was demonstrated by the discrepancy between resident self-report and caretaker report of the provision of tobacco counseling. While control residents reported an increase in the frequency of counseling regarding the risks of passive tobacco exposure, this was not supported by the caretakers' reports. In contrast, the self-reported improvements in SCC behaviors that occurred in the intervention residents were confirmed by both parent report and by chart review and documentation. Therefore, this educational intervention proved successful in changing the SCC behaviors of pediatric resident physicians and we believe that the changes can be directly attributed to the intervention itself.

A unique aspect of this study was the component of counseling validation through chart audits. The educational intervention stressed recording the "stage of change" of the smoker and a quit date if one had been agreed upon. This documentation facilitates patient-centered counseling at future visits, which has been shown to increase the likelihood of moving a patient toward smoking cessation[16]. The residents who received the intervention got this message, as they were more than twice as likely to document "stage of change" of the smoker as compared to controls. One could argue that lack of documentation of counseling and identification of smokers "stage of change" does not necessarily mean that it was not done, but if recorded, it is much more likely that such counseling actually occurred. In addition, documentation allows the possibility of patient-centered advice on follow-up visits.

One can speculate as to why this particular intervention was successful. The didactic educational program used in this study utilized concise and available materials and was taught by one of the general pediatric continuity clinic preceptors. The entire intervention was quite brief, accomplished over a period of 1.5 h. The teaching sessions were conducted at required preclinic conference times when the residents had been relieved of other responsibilities and makeup sessions were easily accomplished when necessary. As a result, the resident participation rates are considerably higher than those reported in other resident educational intervention studies[11,12,13]. We believe that these rates reflect that the intervention was brief, easily available, and administered by someone familiar to the residents. We hypothesize that our residents were receptive to the intervention because it had been designed and implemented in accordance with their continuity clinic practice and its particular resident and patient populations. We conclude that it was the residents' attendance at teaching sessions coupled with their motivation and willingness to learn (and then use) SCC techniques, in addition to the clinic reminders and patient education materials placed in the clinic, that led to the success of this educational intervention.

Finally, we demonstrated that this intervention significantly improved the residents' confidence in SCC skills. Since confidence and prior training have been shown to be positive predictors of SCC in pediatric practice[4], there is hope that these changes will results in lifelong practice habits.

### LIMITATIONS

In this study, residents who attended continuity clinic on three of the five half-days were arbitrarily assigned to receive the SCC intervention and residents who attended clinic on the two remaining half-days were not. This resulted in unequal-sized experimental and control groups. Because the intervention group was larger, there was a greater chance of detecting changes within this group of residents following the intervention. In addition, the group assignments were not random, therefore raising the possiblility of inherent group differences not dirrectly related to the intervention despite the fact that baseline knowledge and attitudes were similar in both the control and intervention groups.

The changes in resident attitudes, behaviors, and confidence in SCC were, in part, measured by self-reports that may not reflect true behaviors/attitudes. The chart audits and follow-up telephone interviews verified that some "reported" behaviors actually occurred. In addition, the residents who did not receive the intervention reported fewer changes in their behaviors, attitudes, or confidence in SCC. This suggests that the intervention was successful at least in the short term. Nonetheless, the inherent inaccuracy of self-reports should be considered when contemplating this study.

Our intent in this study was to obtain follow-up telephone interviews on all 188 participating caretakers who were smokers to ascertain whether they had received SCC during their visit. Only 52% of the caretakers agreed to a follow-up phone call during their visit. One could speculate as to why this was not higher. Possibly it was due to poor communication between physicians and caretakers. Another possibility is the fact that when surveyed, only half of the caretakers saw themselves as nonsmokers in 5 years and, therefore, may not have been interested in phone follow-up regarding their tobacco use. Though we were able to interview 42% of the caretakers who were smokers to complete the survey, it is a minority of the smoking participant group. This should be considered in the interpretation of the results.

### CONCLUSION

In conclusion, this study showed that a brief educational intervention, coupled with the use of clinic reminders and patient educational materials, significantly improved pediatric continuity clinic residents' short-term behaviors and confidence in counseling parents regarding smoking cessation. Future studies will need to address the effects of teaching SCC in pediatric residency programs with regard to long-term changes in physician counseling behaviors and their ultimate effect on caretaker smoking behaviors and quit rates.

### ACKNOWLEDGMENTS

This work was supported by a grant from The University of Kentucky's Children's Miracle Network Telethon Fund.

### BIOSKETCHES

**Rebecca L. Collins, MD**, Department of Pediatrics, University of Kentucky, Kentucky Clinic, J 440, Lexington, KY 40536-0284. Tel: (859) 323-6426, ext. 307. FAX: (859) 257-7706. E-mail: rlcoll2@uky.edu

**Sandy D'Angelo, PhD**, Behavioral Sciences, the University of Kentucky Chandler Medical Center, Lexington, KY.

**Sarah D. Stearns, PhD**, Behavioral Sciences Department, La Rabida Children's Hospital, Chicago, IL.

**Lynn R. Campbell, MD,** Department of Pediatrics, University of Kentucky, Kentucky Clinic, J 430, Lexington, KY.
